# Movement smoothness matters: The missing piece in the functional assessment of rotator cuff patients. Insights from a stereophotogrammetry experimental study

**DOI:** 10.1002/jeo2.70580

**Published:** 2026-03-03

**Authors:** Letizia Mancini, Lorenzo De Sanctis, Emiliano Schena, Pieter D'Hooghe, Alessandro de Sire, Matilde Mancuso, Ara Nazarian, Arianna Carnevale, Umile Giuseppe Longo

**Affiliations:** ^1^ Fondazione Policlinico Universitario Campus Bio‐Medico Roma Italy; ^2^ Research Unit of Measurements and Biomedical Instrumentation Università Campus Bio‐Medico di Roma Roma Italy; ^3^ Research Unit of Orthopaedic and Trauma Surgery, Department of Medicine and Surgery Università Campus Bio‐Medico di Roma Roma Italy; ^4^ Fortius Clinic London UK; ^5^ Department of Medical and Surgical Sciences University of Catanzaro ‘Magna Graecia’ Catanzaro Italy; ^6^ Research Center on Musculoskeletal Health, MusculoSkeletalHealt@UMG University of Catanzaro “Magna Graecia” Catanzaro Italy; ^7^ Carl J. Shapiro Department of Orthopaedic Surgery and Center for Advanced Orthopaedic Studies Beth Israel Deaconess Medical Center Boston MA USA

**Keywords:** biomechanics, kinematics, rotator cuff, shoulder, smoothness

## Abstract

**Purpose:**

This study evaluated whether movement smoothness metrics can effectively distinguish between pathological and healthy upper limbs in patients with rotator cuff tears (RCT), and aimed to identify the most reliable metrics across time and frequency domains. It also investigated the task dependence of movement smoothness across different planes of arm elevation.

**Methods:**

Thirty‐three patients with unilateral RCT performed bilateral synchronous arm elevation tasks in sagittal, scapular, and frontal planes. Movement smoothness was quantified using metrics such as log dimensionless squared jerk (LDSJ), mean arrest period ratio (MAPR) and spectral arc length (SPARC, SPAL), obtained using a 3D Optoelectronic motion capture system. The coefficient of variation (CoV) was used to assess the inter‐subject variability. Differences between limbs were tested with the Mann–Whitney *U* test; task dependency was evaluated with the Wilcoxon signed‐rank test.

**Results:**

Pathological limbs consistently showed lower smoothness than healthy ones across all planes, with SPAL, SPARC, DSJ and LDSJ significantly differentiating the two sides (*p* < 0.05). SPARC and MAPR exhibited the lowest inter‐subject variability, supporting their validity. Smoothness was also task‐dependent, with frontal plane movements being significantly smoother than those in the sagittal or scapular planes (*p* < 0.001).

**Conclusion:**

Smoothness metrics can effectively differentiate pathological from healthy limb movements in patients with RCT, with variability across movement planes. Among the tested metrics, SPARC emerged as the most robust and consistent measure, supporting its use as a biomechanical biomarker for functional assessment and clinical decision‐making.

**Level of Evidence:**

Level II.

**Abbreviations**
AA: angulus acromialis
AC: acromioclavicular joint
AI: angulus inferior
CM: correlation metric
DoF: degree of freedom
DSJ: dimensionless squared jerk
HS: healthy side
IJ: Incisura jugularis
LDSJ: log dimensionless squared jerk
LE: lateral epicondyle
MAPR: movement arrest period ratio
ME: medial epicondyle
OMC: optical motion capture system
OMCs: optical motion capture systems
PC: processus coracoideus
PS: pathological side
PX: processus xiphoideus
RC: rotator cuff
SM: speed metric
SPAL: spectral arc length (alternative implementation)
SPARC: spectral arc length
TS: trigonum spinae
US: ulnar styloid
VAL: velocity arc length

## INTRODUCTION

Shoulder musculoskeletal disorders are one of the most common causes of pain and disability, impacting patients' ability to perform daily activities and reducing their quality of life [8]. Due to the shoulder's inherent complexity, assessing movement quality in patients with musculoskeletal disorders still poses challenges, particularly in the common clinical examination [5, 20]. Clinicians require accurate and reliable features to evaluate movement‐related impairments, plan treatment, evaluate its efficacy, and monitor patients' progress [13, 15, 20].

The 3D kinematic analysis of the shoulder joint with optical motion capture systems (OMCs) is considered the gold standard for obtaining objective data on the characteristics and quality of movement [14]. Indeed, 3D kinematic assessments through OMCs allow for estimating qualitative (e.g., smoothness) and quantitative (e.g., range of motion [ROM]) information on movement performance [22].

Over the years, several kinematic metrics, defined in both time and frequency domains, have been proposed to quantify movement smoothness [4, 9]. Smoothness metrics enable the assessment of small changes in kinematic properties and could enrich traditional clinical evaluations, offering additional insights for assessing movement quality [4].

Most human motor disorders introduce irregularities in movement trajectories and velocity profiles, resulting in less smooth movements [4]. Several studies have discussed the strengths and weaknesses of smoothness metrics, such as their sensitivity to noise, independence from movement amplitude and duration, reproducibility, and interpretative complexity [3, 18, 22].

A recent review proposed a selection of smoothness metrics in terms of validity [3] and robustness [18], namely, speed metric (SM), mean arrest period ratio (MAPR), correlation metric (CM), dimensionless squared jerk (DSJ), and log dimensionless squared jerk (LDSJ), velocity arc length (VAL) in the time domain, as well as spectral arc length (SPARC or SPAL) in the frequency domain [18]. Moreover, the effect of the arm side and the plane of elevation on smoothness was demonstrated in healthy volunteers [21].

According to current literature, smoothness metrics are widely used to assess the movement quality of the upper limb during reaching tasks among patients with neurological conditions such as multiple sclerosis, hemiparesis in children, and acute stroke [9]. These metrics have also been employed in the sports field to assess athletes' coordination and motor skills [1]. Despite their relevance in assessing movements' quality, the evaluation of smoothness in patients with musculoskeletal disorders – especially those affecting the shoulder – remains underexplored.

Among shoulder musculoskeletal disorders, rotator cuff (RC) diseases are a significant cause of diminished quality of life, associated with pain and limited functionality [11, 12, 21]. Smoothness evaluation in patients with RC diseases could be valuable in understanding movement quality and patterns, aiming at identifying new features for monitoring recovery or tailoring treatments.

The main objective of this study was to evaluate movement smoothness using several metrics in both time and frequency domains while performing bilateral synchronous arm elevation tasks in the sagittal, scapular, and frontal plane in patients with RC tears. The main hypothesis was that the smoothness of movement would differ between the pathological and healthy limb. This difference was expected to vary depending on the plane of elevation [20]. Furthermore, it was hypothesised that the pathological side would exhibit reduced smoothness compared to the healthy side.

## METHODS

### Study design and participants

For this cross‐sectional study, 33 patients were consecutively recruited from the Department of Orthopedic and Traumatology at the Fondazione Policlinico Universitario Campus Bio‐Medico. The study was approved by a local Ethics Committee (protocol code: 15.1(21).21 OSS ComET UCBM), and informed consent was obtained from all participants before data collection. Eligibility for inclusion required participants to have a full‐thickness tear of the supraspinatus tendon confirmed by magnetic resonance imaging, no prior surgical interventions on the affected shoulder, no radiographic evidence of fractures in either the glenoid fossa or the greater or lesser tuberosities, absence of any historical shoulder instability, and age over 18. Conversely, exclusion criteria included refusal or inability to provide informed consent, previous surgical treatments of the shoulder, fractures, pseudoparalytic or stiff shoulders or additional pathological conditions in the affected shoulder, such as involvement of the long head of the biceps brachii. Demographic and clinical data, including sex, age, anthropometric measurements, limb dominance and pathological limb, were collected before each trial. Additionally, MRI was also performed on the contralateral shoulder to exclude the presence of asymptomatic rotator cuff tears.

For detecting the difference between the pathological and healthy sides, with an effect size of 0.7, a significance level of 5%, and 80% power, a sample size of 32 patients was required. The effect size was computed using the Cohen's index, considering the mean values and standard deviations of the SPARC reported by Bayle et al. for the dominant and non‐dominant sides in healthy volunteers. These values were chosen because there are no similar studies in the literature focusing on shoulder musculoskeletal disorders. The sample size calculation was performed using G*Power (v. 3.1, Erdfelder, Faul, & Buchner) [6].

### Data collection

**Figure 1:**
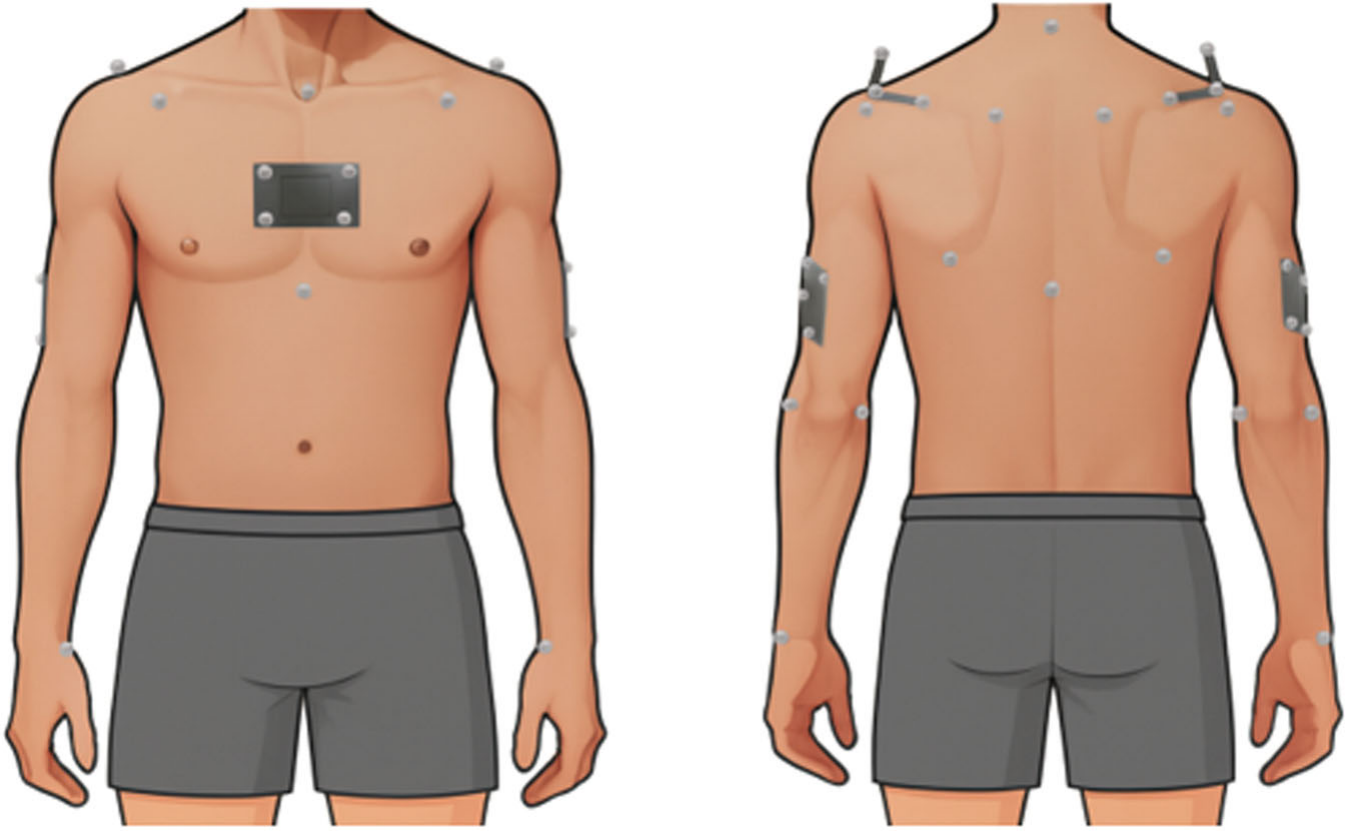
Placement of anatomical markers and clusters on the subject's body.

Spherical retroreflective markers (diameter: 8 mm) were placed onto the Incisura Jugularis (IJ), Processus Xiphoideus (PX), C7 and T8 vertebra landmarks, Angulus Acromialis (AA), Trigonum Spinae (TS), Angulus Inferior (AI), Acromioclavicular joint (AC), Processus Coracoideus (PC), medial (ME) and lateral (LE) epicondyles of the humeri. Two markers were placed laterally on the right and left ulnar styloid (US), respectively, after palpation. A rigid cluster, consisting of four markers, was fixed with double‐sided medical tape on the sternum, midway between IJ and PX. Other two clusters were fixed with elastic bands on the right and left upper arm in the middle third, laterally, slightly distal. Two L‐shaped clusters, consisting of three markers each, were placed with double‐sided medical tape on the acromion plateau of the two scapulae (Figure 1). All the markers were tracked while collecting data from the OMC system (Qualisys AB, Göteborg, Sweden) [25].

After the static calibration recording in the neutral pose (arms neutral besides the body), patients were asked to perform three elevation tasks bilaterally, keeping the elbow fully extended: (i) shoulder elevation in the sagittal plane (forward flexion), (ii) shoulder elevation in the scapular plane (scaption) and (iii) shoulder elevation in the frontal plane (abduction). All tasks were performed five times, with patients executing the movements up to the maximum pain‐free range of motion they could achieve, at a comfortable and self‐selected speed. All patients practiced a supervised trial of the tasks before each recording [10].

### Data analysis

All tasks were uniformly sampled at 100 Hz. The marker trajectories were manually gap‐filled in QTM (version 2024.1, Qualisys AB, Göteborg, Sweden), and a zero‐lag, fourth‐order Butterworth filter with a cutoff frequency of 6 Hz was applied. The preprocessed data were then exported for kinematic and statistical analysis in MATLAB (version R2024a, MathWorks Inc., Natick, MA, USA).

The glenohumeral joint was modelled as a ball‐and‐socket joint with 3 degrees of freedom (DoF). The elbow was modelled as a fixed joint. Acromioclavicular and sternoclavicular joints were not considered. The thorax TH frame was defined as per ISB guidelines and tracked with the associated cluster [25]. The humeral H frame was defined as the ISB‐recommended ‘H1’ and tracked by the cluster placed on the upper arm [25]. The glenohumeral joint centre was estimated by linear regression using the scapula landmarks and tracked by the acromion marker cluster [16, 24]. Joint angles were calculated by Euler decomposition with the YXY sequence, using H frame with respect to TH. The 3D trajectories of the ulnar styloids were transformed with respect to the TH reference frame, and their vertical y‐axis component was collected. For each patient, for each side, and for each task, the ascending phase of the three central repetitions of the time series was considered. All the time series were automatically segmented in MATLAB, taking as the starting point the last instant whose US marker velocity is less than 5% the maximum calculated over the entire task trajectory [19]. The indices for window extraction were found on the trajectory of the healthy limb, and the movement of both limbs was assumed to be synchronous, as instructed by the operator at recording. A manual check was conducted on the segmented trajectories to verify that the elevation phases between the two limbs actually matched. All metrics classified as robust in Refai et al. were computed for each time series using the MATLAB codes provided by the authors, including four variants of the dimensionless squared jerk (DSJ), i.e., SM, MAPR, CM, VAL, SPAL and SPARC [18]. The DSJ and its logarithm (LDSJ) were computed using the Teulings method (DSJ_t_, LDSJ_t_), as well as with the Balasubramaniam method (DSJ_b_, LDSJ_b_) [2, 18, 23]. All other analysis code was developed by the authors.

### Statistical Analysis

All calculated smoothness metrics were classified as either healthy or pathological based on the limb from which the time series were segmented.

The Shapiro–Wilk test was carried out to check the data distribution's normality [17]. Considering the data's non‐normal distribution, non‐parametric tests with a 95% confidence interval (p = 0.05) were applied.

The Mann–Whitney test [17] was performed for each metric to determine whether statistically significant differences existed between the pathological limb and the contralateral healthy limb. Additionally, the Wilcoxon signed‐rank test was conducted across different tasks to evaluate task dependency. The statistical significance level was set at p<0.05.

Coefficients of variation (CoV), defined as the ratio of the standard deviation to the mean [4], were calculated between subjects to estimate the variability across individuals for each metric. The analysis was therefore performed on a total of 99 pairs of time series for each metric.

## RESULTS

A total of 14 men and 20 women were analysed, with a mean age of 60.9 ± 8.0 years, a mean height of 1.66 ± 0.09 m, and a mean weight of 75.3 ± 15.5 kg. All participants successfully completed the experimental tasks.

### CoV across arm elevation tasks

The analysis of CoV across various smoothness metrics in different arm elevation tasks, summarised in Table 1, revealed considerable between‐subject variability in both the time and frequency domains.

**Table 1:** Coefficient of variation (CoV) calculated between subjects and Wilcoxon rank‐sum test (*p*‐value) for all the smoothness metrics between HS and PS.

		SM	MAPR	VAL	CM	DSJ_t_	LDSJ_t_	DSJ_b_	LDSJ_b_	SPAL	SPARC
Forward flexion	PS	26	13	207	305	199	24	346	23	36	42
	HS	20	6	97	330	190	23	325	24	16	9
	*p*	<0.001*	<0.001*	0.17	<0.001*	0.01*	0.01*	0.02*	0.02*	0.001*	<0.001*
Scaption	PS	20	7	65	86	193	21	289	21	18	21
	HS	18	4	44	197	132	19	248	19	9	10
	*p*	0.07	0.015*	0.558	0.79	0.034*	0.034*	0.052	0.052	0.03*	0.007*
Abduction	PS	20	10	54	94	113	22	225	23	26	28
	HS	19	8	44	287	103	20	214	22	7	8
	*p*	0.35	0.46	0.28	<0.001*	0.002*	0.002*	0.002*	0.002*	<0.001*	<0.001*

Abbreviations: HS, healthy side; PS, pathological side.

* Statistical significance (*p* < 0.05).

In forward flexion, SM showed a CoV of 26% on the PS and 20% on the HS, MAPR exhibited 13% on the PS and 6% on the HS, SPAL showed 36% on the PS and 16% on the HS, and SPARC recorded 42% on the PS compared to 19% on the HS.

During scaption CoVs of 7% on the PS and 4% on the HS for MAPR, and 21% on the PS and 10% on HS for SPARC. The LDSJ_t_ and LDSJ_b_ metrics show differences of 21% in PS and 19% HS for LDSJ_t_ and 21% vs 18% for LDSJ_b._

During abduction, LDGS_t_ showed CoVs of 22% on PS and 20% on HS, SPAL 26% on PS and 7% on HS, and SPARC with 28% on PS and 8% on HS.

### Smoothness metric comparison between pathologic and healthy side

In forward flexion, all metrics showed significant differences (p<0.05), except for VAL. For scaption, the MAPR, SPAL and SPARC metrics displayed significant differences, while the CM, SM, and VAL did not. In abduction, the CM, SPAL and SPARC metrics reached statistical significance (p < 0.001), whereas SM, VAL and MAPR did not. Moreover, all jerk‐related metrics, including the squared and logarithmic dimensionless jerk (computed using both Teulings and Balasubramaniam methods), showed statistically significant differences between the pathological limb and the contralateral limb (p<0.001).

### Task‐dependent variability

Table 2 shows significant differences in smoothness metrics across different arm‐elevation planes, both in the pathological and the healthy side. These significant differences emphasise the task nature's considerable impact on movement smoothness.

**Table 2:** Wilcoxon signed‐rank test *p*‐values for all the smoothness metrics between different task.

		SM	MAPR	VAL	CM	DSJ_t_	LDSJ_t_	DSJ_b_	LDSJ_b_	SPAL	SPARC
**Sagittal plane/scapular plane**	PS	<0.001*	<0.001*	<0.001*	<0.001*	<0.001*	<0.001*	<0.001*	<0.001*	0.009*	0.01*
	HS	<0.001*	0.002*	0.003*	<0.001*	<0.001*	<0.001*	<0.001*	<0.001*	0.074	0.01*
**Frontal plane**/**sagittal plane**	PS	<0.001*	0.006*	0.003*	<0.001*	<0.001*	<0.001*	<0.001*	<0.001*	0.01*	<0.001*
	HS	0.14	0.02*	0.038*	0.12	<0.001*	<0.001*	<0.001*	<0.001*	<0.001*	<0.001*
**Frontal plane**/**scapular plane**	PS	0.008*	0.004*	0.98	0.27	0.85	0.44	0.52	0.11	0.41	0.27
	HS	<0.001*	<0.001*	0.688	<0.001*	0.017*	0.004*	0.002*	<0.001*	<0.001*	0.001*

* Statistical significance (*p* < 0.05).

In the comparison between the sagittal and scapular planes, the pathological side shows significant differences all metrics – SM, MAPR, VAL, CM, DSJ_t_, LDSJ_t_, DSJ_b_, LDSJ_b_, SPAL and SPARC – with *p*‐values below 0.05. A similar trend of significant differences is observed on the healthy side for all metrics, except for SPAL which did not reach statistical significance (*p >* 0.05).

In the frontal versus sagittal plane comparison, the pathological side reveals substantial differences with significant *p*‐values less than 0.001 for SM, MAPR, VAL, DSJ_t_, LDSJ_t_, DSJ_b_and LDSJ_b_. The healthy side, while showing less pronounced differences, still presents significant findings in DSJ_t_, LDSJ_t_, DSJ_b_, LDSJ_b_, SPAL and SPARC, with MAPR and VAL also demonstrating significant differences (*p <* 0.05).

Conversely, the comparison between the frontal and scapular planes on the pathological side does not exhibit significant differences, indicating a uniform response across these task orientations. However, the healthy side shows significant differences in all metrics, except for VAL, with *p <* 0.001.

## DISCUSSION

This study assessed 10 smoothness metrics derived from both time and frequency domains in patients with RCT during bilateral synchronous arm elevation tasks across sagittal, scapular and frontal planes.

**Figure 2:**
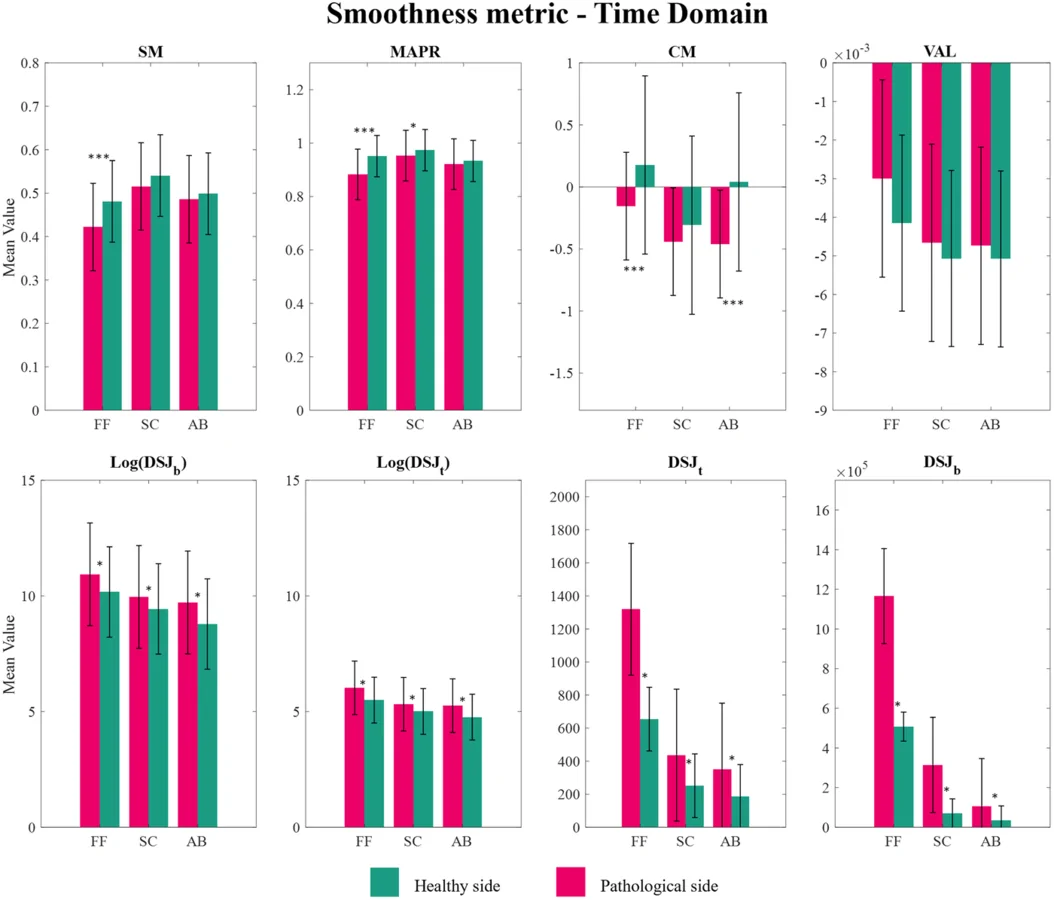
Time–domain smoothness metrics for healthy and pathological limbs across different movement tasks. AB, abduction; FF, forward flexion; SC, scaption. *p < 0.05, ***p < 0.001.

**Figure 3:**
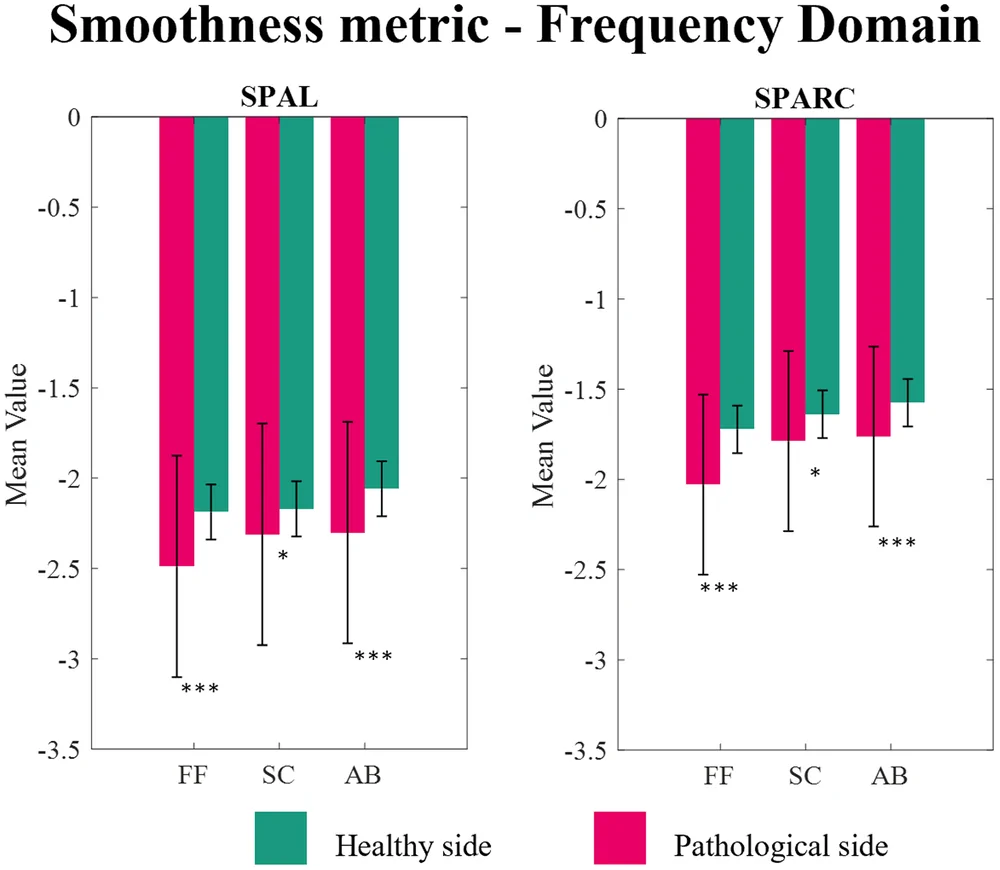
Frequency–domain smoothness metrics for healthy and pathological limbs across different movement tasks. AB, abduction; FF, forward flexion; SC, scaption. *p < 0.05, ***p < 0.001.

In accordance with our initial hypothesis, significant differences were observed between the PS and the HS across several smoothness metrics in both time and frequency domains (Table 1, and Figures 2 and 3), highlighting their ability to distinguish between pathological and healthy conditions. SPAL (*p* ≤ 0.001) and SPARC (*p* ≤ 0.001) consistently showed significant differences during FF and AB, aligning with previous findings by Bayle et al., who demonstrated SPARC's robustness in discriminating limb‐specific motor characteristics in healthy middle‐aged subjects [4]. Jerk metrics (e.g., LDSJ_t_, LDSJb; *p* < 0.05) consistently showed significant differences, similar to those reported by Roren et al. and Bayle et al., who also found significant differences in LDSJ between the dominant and non‐dominant limbs in healthy subjects [4, 20]. Additionally, SM demonstrated significant differences between PS and HS during forward flexion (*p* < 0.001), consistent with findings by Gulde et al., who applied this metric to compare healthy young and elderly subjects [7].

Our analysis identified SPARC and MAPR as metrics with the lowest inter‐subject variability, showing consistently lower CoV in HS, as shown in Table 1. This aligns with findings from Bayle et al., who similarly recognised SPARC as a valid smooth metric due to their lower between‐subject variability (SPARC CoV: 1.6%–3.5%) followed by the LDLJ (with higher CoV: 6.8%–8.7%) [4]. Conversely, metrics such as VAL, CM, LDSJ_t_ and LDSJ_b_, exhibited higher levels of inter‐subject both in PS and HS, indicating that these metrics might be less suitable for direct comparisons across experimental conditions, unless other kinematic parameters (e.g., movement duration, amplitude and speed) are strictly controlled, as also suggested by previous studies [4, 18].

The analysis of intersubject variability also showed wide differences between the various jerk metrics, dependent on their formulation. Specifically, according to our results reported in Table 1, the LDSJ_t_, showed the best performance in terms of discriminative ability (statistically significant differences with *p* < 0.05 in all three tasks) and validity (lower CoV).

The comparison of smoothness metrics across different arm elevation tasks (Table 2, and Figures 2 and 3) revealed significant task‐dependent variability, with frontal plane movements being smoother than sagittal and scapular ones. Significant differences (*p* < 0.001) were observed in all metrics when comparing sagittal versus scapular planes (e.g., SM, MAPR, VAL, CM, LDSJ_t_, DSJ_t_, LDSJ_b_ and DSJ_b_), as well as frontal versus sagittal planes. While our findings align with Roren et al. regarding the task‐dependent nature of movement smoothness, they differ in the plane where movements are most smooth [20]. In fact, contrary to Roren et al., who reported smoother movements in the sagittal plane, our data indicate that movements in the frontal plane exhibit greater smoothness in patients with RTC. This discrepancy may reflect compensatory strategies or pain avoidance mechanisms specific to RCT pathology. The task‐dependence observed in our study highlights that smoothness is a context‐sensitive property of the movement. The observed variations across tasks also emphasise the potential for smoothness metrics to provide task‐specific biomarkers, useful for individualized rehabilitation planning.

To the best of our knowledge, this is the first study to quantify and compare multiple smoothness metrics during bilateral synchronous arm elevation tasks across sagittal, scapular, and frontal planes in patients with RCT. The use of well‐established metrics from both time and frequency domains enables a comprehensive evaluation of movement quality, providing features that enhance their suitability for both clinical and research applications. Furthermore, the inclusion of a task‐specific analysis provides novel insights into the task‐dependency of smoothness in shoulder disorders. Additionally, comparing the four jerk formulations enriches the methodological value, offering guidance for metric selection in future research.

This study represents the first examination of these metrics in patients with RCT. It aims to assess their relevance for individuals with these pathologies, extending the focus beyond earlier research that primarily investigated healthy individuals, as well as those with Parkinson's disease and stroke.

Smoothness can contribute to real time clinical assessment as a complementary marker of movement quality. A task‐specific smoothness index reported together with range of motion and symptoms can provide immediate feedback during standardised elevation tasks and support diagnostic reasoning by highlighting compensatory strategies that may not be evident when range of motion and pain appear comparable. The same index can be tracked across visits to characterise post‐treatment change and to inform progression of exercise.

Beyond the clinical context, the analysis of movement smoothness may also offer valuable insights within sports performance analysis [9]. Its integration into return to play decision pathways should be explored cautiously as an adjunct to strength, range of motion and symptom criteria. These perspectives are promising yet require prospective work that harmonises protocols, quantifies minimal detectable change and minimal clinically important difference, and links changes in smoothness to clinically meaningful outcomes. Framed in this way, smoothness offers a realistic path to becoming an actionable descriptor of shoulder movement quality while remaining complementary to established clinical measures.

Although our study provides valuable insights, it has some limitations. Primarily, the absence of a follow‐up prevents evaluating the metrics' sensitivity to treatment outcomes or progression over time. Patients were instructed to perform each task at their preferred speed. Including trials at varying, imposed velocities – such as slow, moderate speeds – could have helped to more thoroughly evaluate the sensitivity of the smoothness metrics to changes in movement duration. Expanding the analysis to include a broader range of daily living activities could help to better understand the generalisability and validity of smoothness metrics.

## CONCLUSION

This study demonstrated that smoothness metrics can effectively differentiate between healthy and pathological shoulder movements. Among the evaluated metrics, MAPR, SPAL, LDSJ, and SPARC exhibited lower inter‐subject variability across all tested movement tasks. Notably, SPARC emerged as the most reliable and consistent, suggesting its potential as a preferred kinematic indicator for monitoring shoulder dysfunction and recovery.

Moreover, our findings clearly demonstrate that smoothness is inherently task‐dependent, reinforcing the importance of selecting appropriate evaluation tasks in clinical assessments. In conclusion, the smoothness metric provides a quantifiable window into movement quality. It could hold promise as a potential biomarker for assessing motor function and monitoring pathological conditions, such as RCT.

## AUTHOR CONTRIBUTIONS

All authors contributed to the conception and design of the study. All authors read, reviewed and approved the final version of the manuscript.

## CONFLICT OF INTEREST STATEMENT

The authors declare no conflicts of interest.

## ETHICS STATEMENT

Ethical approval for this study was granted under the protocol code: 15.1[21].21 OSS ComET UCBM. Informed consent was obtained from all participants included in the study. The participants were provided with comprehensive information about the purpose of the research, the procedures involved, and any potential risks. They were also informed of their right to withdraw from the study at any time without consequence. All participants gave their written consent for their data to be used in this research and for any publication of the results.
